# Notes on Preparations for the Sick

**Published:** 1909-09

**Authors:** 


### Notes on Preparations for the Sick.

**Soloid Products for Ionic Medication.**—BURROUGHS, WELLCOME & Co., London.—The old method of treatment known as cataphoresis or ionic medication has been recently reintroduced, and is recognised to have a distinct field of usefulness. It is finding increasing vogue in this and other countries. The treatment consists essentially in the electrolytic dissociation of drugs, the dissociated ions having the power of penetrating the skin and reaching the deeper tissues. Drugs intended for absorption applied in the form of liniments or ointments penetrate the skin with difficulty, but by means of electrolysis they can be introduced with comparative ease.

The following is a list of soloids prepared: Cocaine Hydrochloride, grs. 4.37; copper sulphate, grs. 4.37; magnesium sulphate, grs. 4.37; potassium iodide, grs. 4.37; sodium chloride, grs. 4.37; sodium salicylate, grs. 4.37; quinine bisulphate, grs. 4.37; zinc sulphate, grs. 4.37.

Each soloid dissolved in one fluid ounce of water gives a 1 per cent. solution.

**Vaporole: Morphine Hydrochloride,** 0.01 in 1 cc.; **Cocaine Hydrochloride,** 0.01 in 1 cc.; **Pituitary (Infundibular) Extract,** 1 cc. = 0.2 (grs. 3).—BURROUGHS, WELLCOME & Co., London.—These vaperoles are small glass capsules, hermetically sealed, with sterilised contents, for hypodermic or intermuscular use. The morphia and the cocaine vaporole contain one sixth of a grain of the drug, ready for immediate use by breaking off the neck of the container.

The pituitary extract causes a rise of blood pressure, due to a general vaso-constriction set up by the stimulation of the unstriped muscle of the arteries. It, therefore, acts as a cardiac tonic in case of shock and as a stimulant to uterine contraction.

Various other vaporoles are prepared containing single doses ready for use of such drugs as ernutin, eucaine, hemisine, hyoscine, cocaine, atropin, calomel and grey oil, &c.

**Lactic Bacillary Tablets** (Fairchild).—Burroughs, Wellcome & Co., London.—These tablets contain a culture of the true Oriental lactic acid bacillus in a convenient form for internal administration.

---

**Tabloids.**—Burroughs, Wellcome & Co., London.—

**Pepana.**—A keratin-coated and sugar-coated disc containing one grain each of pancreatin and calcium lactophosphate enveloped with pure pepsin. They should be very efficient as a gastro-enteric digestive.

**Calcium Iodo-Ricinoleate,** grs. iij.

**Phenol and Menthol Compound.**—Phenol, gr. $\frac{1}{2}$; menthol, gr. $\frac{1}{2}$; Ol. cajuputi, gr. j.

**Laxative Fruit Pastilles.**—These pastilles have been found to be quite efficient.

---

**Sagrada Barber.**—Wilcox, Jozeau & Co., London.—Sagrada Barber is a pure dry extract of Cascara Sagrada from the bark of rhamnus purshiana. It includes the bitter principle of the bark, such principle having proved to be an excellent bitter-tonic.

These purgative pastils are oval-shaped; they are coated with a thin, readily soluble layer of the finest cocoa, and, in consequence are non-hygroscopic, keep for a long time, and are perfectly odourless and tasteless. Each pastil weighs 0.50 gramme, corresponding to 3·0 grammes of liquid extract.

---

**Veronal-Sodium ; Sabromin.**—The Bayer Company, Elberfeld and London.—Veronal-Sodium, as the name suggests, is a modification of the well-known hypnotic veronal. While it is not likely to replace the latter, it is more convenient to use in that it is readily soluble (1 to 6) in cold water, whereas hot liquid is necessary to dissolve the older preparation. Its indications are, of course, similar, viz. all conditions of sleeplessness, unless those associated with severe pain, and it is specially advised where it is found necessary to administer a hypnotic per rectum.

The dose is five to fifteen grains, given half an hour before bedtime. As with veronal, it is always best to commence with the minimum dose

Sabromin bears the same relation to the bromides that sajodin does to the iodides. The calcium salt of di-bromo-behenic acid is a white odourless and tasteless powder. While, as compared with potassium and the other bromides it is equally efficacious, it is at the same time more easily taken, does not irritate the digestive organs, has far less tendency to produce bromism, and is especially adapted for continued administration.

The dose is five to fifteen grains, thrice daily after food, and may be taken dry on tongue or in the form of cachets or tablets (crushed before taking) followed by a draught of water.

**Thyresol.**—THE BAYER COMPANY, Elberfeld and London.—A new sandalwood oil preparation. It is chiefly remarkable in that, while it has powerful antiseptic properties quite equal to those of santalol, the latter body is not liberated in the system. Thyresol contains no free hydroxyl groups, and is relatively non-toxic. Mainly for these reasons it obviates such unpleasant by-effects so frequently associated with sandalwood oil medication, as eructation, irritation of the stomach, loss of appetite.

The dose of thyresol is five to ten grains three or four times a day, and may be taken either in drops, capsules or tablets. A feature of the last-named is the magnesia carbonate forming their base, the combination exerting a distinct laxative effect—an obvious advantage in gonorrhœa.

---

**Bromoglidine.**— MENLEY & JAMES, London.—We have already called attention to glidine (p. 183), a concentrated protein food manufactured wholly from wheat, and iodoglidine, an organic combination of iodine with vegetable protein.

Many trials of these preparations have testified to the excellent results which have followed their administration. Glidine has proved of great value as an effective tonic-nutrient in cases of diabetes, anæmia, phthisis, and wasting disorders ; whilst remarkable successes have been achieved with iodoglidine in the treatment of arterio-sclerosis, rheumatoid-arthritis, asthma and gout.

A bromide compound, in tablets containing 0.05 grain bromine in organic combination with the vegetable protein, has now been introduced, and should prove itself of much value in various disorders of the nervous system.

---

**Laxol.**—MENLEY & JAMES, London.—Laxol is simply the purest cold-drawn castor oil that can be obtained, to which small quantities of oil of peppermint, vanillin and other flavouring and sweetening agents are added. We specially emphasise the fact that no medicinal agent whatever has been added to the oil, nor is the oil in the form of an emulsion. The nauseous flavour of the oil is not only entirely overcome, but the oil is actually rendered quite palatable, especially to children. Indeed, it has been said to be as sweet as honey.

We have reason to believe that laxol is largely, if not altogether, free from the after constipative effects of ordinary castor oil.

---

**Biogene.**—BONTHRON & CO., London.—Any new variety of food for the diabetic should be cordially welcomed. Biogene is a soluble casein, rich in phosphates, containing about 90 per cent. of proteid, absolutely free from sugar and starch, is easy of digestion, is almost tasteless, it can be added readily to any ordinary food to improve its nutrient value, and is put up in the convenient form of biscuits, which should be acceptable and useful not only in cases of diabetes, but in many other conditions of general debility.

---

**Sumner's Covers.**—R. Sumner & Co., Liverpool.—These simple covers are intended to be used for vessels containing milk, sugar and other foods likely to be contaminated by flies. For several years the medical profession have been vigorously advocating sterilised and pure milk as a means of preventing summer diarrhœa in infants. But notwithstanding that these methods are now very frequently adopted, infantile diarrhœa is very prevalent.

In an extract of his M. D. Thesis, published in the *Lancet* of September 5th, 1908, Dr. V. J. Glover proves that the house fly is often the carrier of infection to milk, and so to infants; also that it conveys infection to the breast-fed infant by entering the child's mouth whilst it sleeps, and depositing the germ in the saliva.

Whilst approving of sterilisation, he contends these precautions are almost useless unless steps are taken to prevent contamination of the milk afterwards by flies. He recommends "suitable covers" to be placed over all vessels containing milk, to prevent the fly reaching and depositing infection therein. This is a very simple but most important method of preventing that terrible disease, infantile diarrhœa; and they have made, with Dr. Glover's approval, simple, cheap, and yet highly efficient coverings for vessels containing milk, as well as gauze covers for the cot, to guard against the fly entering the child's mouth and depositing infection in the saliva.

